# The association between substance use and common mental disorders in young adults: results from the South African Stress and Health (SASH) Survey

**DOI:** 10.11694/pamj.supp.2014.17.1.3328

**Published:** 2014-01-18

**Authors:** Amina Saban, Alan J Flisher, Anna Grimsrud, Neo Morojele, Leslie London, David R Williams, Dan J Stein

**Affiliations:** 1 Adolescent Health Research Unit, Department of Psychiatry and Mental Health, University of Cape Town, Cape Town, South Africa; 2 School of Public Health, University of Cape Town, Cape Town, Cape Town, South Africa; 3 Alcohol and Drug Abuse Research Unit, Medical Research Council, Pretoria, South Africa; 4 Harvard University School of Public Health, Boston, USA; 5 Department of Psychiatry and Mental Health, University of Cape Town, Cape Town, South Africa

**Keywords:** SASH, comorbidity, mental disorders, substance use

## Abstract

**Introduction:**

Although substance use is commonly associated with mental disorders, limited data on this association are available from low and middle income countries such as South Africa. The aims of the study were i) to determine patterns of substance use in young adults, ii) to identify trends of common psychiatric disorders in relation to use of specific substances, and iii) to determine whether specific psychiatric disorders were associated with use of specific substances in the South African population.

**Methods:**

Data were drawn from the South African Stress and Health (SASH) study, a nationally-representative, cross-sectional survey of South African households that forms part of a World Health Organisation (WHO) World Mental Health (WMH) initiative to standardise information on the global burden of mental illness and its correlates. Data from a subset (n = 1766; aged 18 to 30 years) of the SASH sample of 4351 individuals were analysed. The Composite International Diagnostic Interview Version 3 (CIDI 3.0) was used to elicit basic demographic details and information regarding mental illness and substance use. Multiple regression analyses, adjusted for age and gender, were used to identify associations between mental disorders and substance use.

**Results:**

Significant associations were found between substance use and mood and anxiety disorders, with a particularly strong relationship between cannabis use and mental disorder.

**Conclusion:**

The results are consistent with those from previous studies, and reinforce the argument that comorbid substance use and mental disorders constitute a major public health burden.

## Introduction

Debilitating mental illness often places a burden on society [1]. Mental illness can result in reduced economic productivity in affected individuals [2], and can influence the quality of life for individuals and their families as a result of the disabling effects of the mental illness [3]. Mental illness can also increase the load on service providers [4]. Similarly, problematic substance use poses a challenge to society because of its effects on the psychosocial functioning, productivity and general health of the affected individuals [5, 6]. It has been argued that individuals who suffer from mental illnesses are more likely to be or become dependent on substances than are individuals who do not have mental disorders [4]. Conversely, individuals who abuse substances appear to be more likely to develop or suffer from mental illnesses than are those who do not abuse substances [7]. Thus, when mental illness and problematic substance use or abuse co-occur, the resulting problems are often compounded and more complex [8], increasing the challenges posed to the management and treatment of the affected individuals [4].

Although many studies have examined comorbid psychopathology and substance use globally, more details on comorbidity are generally available for older individuals, and less regarding children, adolescents and young adults [6]. Furthermore, most of these studies have examined samples in treatment for either substance use or psychiatric disorders [9], resulting in less published information on comorbidity in communities.

Armstrong and Costello [9] described six important reasons for examining comorbid psychopathology and substance use in representative community samples. Firstly, since individuals with more than one disorder are more likely to seek treatment for either disorder than are individuals with only one disorder, clinical samples are more likely than community samples to contain comorbid individuals. Secondly, the nature of some disorders might be more likely to precipitate treatment seeking. Hence, for example, a disruptive behaviour disorder (such as conduct or antisocial personality disorder) that co-occurs with substance use might be more likely to elicit treatment seeking than would an internalising disorder (such as depression), resulting in clinical samples possibly having a higher percentage of comorbid disruptive behaviour disorders than is commonly prevalent in communities. Thirdly, community and clinical samples might differ with respect to the severity of occurring symptoms. Fourthly, community and clinical samples might differ with respect to the temporality of comorbidity. Fifthly, clinic and community samples might differ with respect to the risk factors for comorbid psychopathology and substance use. These differences can usually be assessed for diseases where most cases are treated. However, since not all comorbid substance users receive treatment, it would be inappropriate to assume that risk factors for comorbidity are the same in both treatment and community samples. Lastly, substance use treatment samples might reflect a different patient economic profile compared with community samples by containing mainly individuals who have access to finances for treatment, and thus excluding those individuals who might need treatment but cannot afford it.

In South Africa, as in other developing countries, information regarding comorbid psychopathology and substance use, its consequences, and the implications for society, is limited by a paucity of published studies [10, 11]. Evidence has indicated that sociodemographic factors can play a significant role in comorbidity and its effects [12–20]. Findings from non-South African studies have included evidence for associations between conduct disorder and substance use within specific developmental stages [21, 22], and differing associations between psychopathology and substance use in males and females [23, 16]. While some findings included evidence for associations between depression and smoking [17, 24, 25] between depression and alcohol use [26], and between psychopathology in general and cannabis use [19], other findings have not indicated definitive associations between psychopathology and substance use [18, 27, 28]. For example, Boyle et al. [27] found associations between conduct disorder and cannabis use, but not between conduct disorder and use of tobacco or alcohol, while Costello et al. [18] found no evidence for associations between anxiety and substance use. Though these findings play a significant role in the understanding of associations between psychopathology and substance use, their generalisability to the South African population cannot be assumed. In particular, these studies had not always used similar psychopathology assessment tools, had differing measurements of substance of use, and subscribed to different conceptual frameworks, suggesting that over-arching conclusions about the nature of comorbid psychopathology and substance use be drawn with caution.

The current study focussed on the nature of associated psychopathology and substance use in a previously unexamined sample of young South African adults aged 18 to 30 years. The study advances knowledge about comorbidity in general, and particularly in South Africa, by using a nationally-representative community sample. The aim of the study was threefold. Firstly, the prevalence of substance use was calculated with respect to selected demographic factors to determine substance use patterns in young adults. Secondly, the prevalence of psychopathology (namely mood and anxiety disorders) amongst younger users of various selected substances was calculated, to determine percentage of psychopathology in relation to specific substances of use. Thirdly, the current study calculated associations between selected lifetime and 12-month psychopathology (namely mood and anxiety disorders) and substance use, adjusted for age and gender to determine whether specific psychopathology was associated with use of specific substances of use in young South African adults.

## Methods

This study draws on the nationally-representative South African Stress and Health (SASH) survey, the South African arm of the World Health Organisation's (WHO) World Mental Health (WMH) initiative, aimed at providing standardised information on the global burden of mental disorders and its correlates in 28 countries [29, 30]. The SASH study was conducted between January 2002 and June 2004 [31]. The study protocol was approved by the Institutional Review Board of the University of Stellenbosch. All sampled individuals provided written informed consent for participation in the study.

### Sample

The SASH study randomly sampled adult males and females, aged 18 years and older, from all the South African racially classified social groups (RCSGs). RCSGs are defined according to the Population Registration Act of 1950 that classified citizens in terms of skin colour, and still reflect social disparities in South Africa [32]. The RCSGs were Asian/Indian, White, Coloured and Black, with Coloured referring to individuals of mixed racial origin. A three-stage probability sample design was used. The primary stage involved stratification by census enumerator areas (EA). A probability sample of households (that excluded prisons, hospitals and military barracks) and hostel quarters (single-sex migrant labour accommodation) was selected from each EA. One adult was randomly selected from each household included in the sample. Of these, questionnaires were adequately completed for 98.1% of the interviewed individuals, resulting in a sample size of 4351 participants. The present study extracted data regarding those individuals who were aged 18 to 30 years (inclusive), to address comorbid psychopathology and substance use in young adults (sub-sample size of 1766).

### Instruments

The mental health of each selected individual was assessed using the lay-administered World Mental Health (WMH) Composite International Diagnostic Interview Version 3 (CIDI 3.0) [33], providing both Diagnostic and Statistical Manual-Version IV (DSM IV) and International Classification of Diseases-Version 10 (ICD-10) psychiatric diagnoses for lifetime and the last 12-months. Since the CIDI had not previously been used in a South African population sample, parallel validation interviews were conducted by a clinician who was blind to the CIDI diagnoses, on 100 study participants [31]. The interview questions were translated from English into six of the 11 official languages in South Africa, to enable respondents to communicate in their mother tongue. Iterative back-translation was conducted by panels of bilingual and multilingual experts following WHO recommendations and discrepancies resolved by an expert consensus panel.

### Procedure

Interviewers were trained to use the CIDI and become fieldworkers, operating in conjunction with fieldwork supervisors to identify and select potential subjects. The scheduled face-to-face interview was completed by the fieldworker, usually at the home of the participant, after informed consent had been obtained from the subject. Interviews were conducted in seven of the 11 official languages of South Africa, namely English, Afrikaans, isiXhosa, isiZulu, seSotho, Northern Sotho and seTswana. The duration of interviews averaged three hours. In the event of an interview not being completed in one sitting, a second interview was scheduled and conducted.

### Measures

The pencil-and paper version of the CIDI was administered to all study participants. It elicited lifetime and 12-month occurrence for major depression (MD), panic disorder, social phobia, agoraphobia, generalised anxiety disorder (GAD), intermittent explosive disorder (IED), suicidality, substance use and post-traumatic stress disorder (PTSD), while personality disorders and psychoses were screened for. Anxiety disorders included panic disorder, generalised anxiety disorder (GAD) with hierarchy, social phobia, agoraphobia, and PTSD. A summary category was created ‘any anxiety disorder or mood disorder. Diagnoses of mood (namely, major depression) and anxiety disorders were selected for analyses in the present study (Table 1, Table 2, Table 3, Table 4), thus excluding intermittent explosive disorder, suicidality and those disorders that were screened for (personality disorders and psychoses).

**Table 1:** Prevalence of substance use by demographics

		Tobacco		Alcohol		Cannabis		Other substances^a^		Non-medical use of prescription drugs b	
		100+ cigarettes (%)	p-value	Ever (%)	p-value	Ever (%)	p-value	Ever (%)	p-value	Ever (%)	p-value
**Total sample**		27.4 (24.9-30.1)		38.7 (35.5-41.9)		10.7 (9.0-12.8)		2.7 (1.8-4.0)		20.6 (17.5-24.1)	
**Gender**	Males	44.9 (40.2-49.8)	<0.001	54.0 (49.8-58.1)	<0.001	17.7 (14.5-21.6)	<0.001	4.2 (2.5-7.0)	0.001	21.4 (17.2-26.4)	0.497
	Females	9.8 (7.5-12.6)		22.7 (19.4-26.4)		3.6 (2.5-5.2)		1.1 (0.6-1.9)		19.8 (16.7-23.4)	
**RCSG**	Black	23.7 (21.2-26.3)	<0.001	34.0 (30.5-37.7)	<0.001	9.3 (7.5-11.4)	<0.001	2.1 (1.3-3.4)	0.004	21.2 (18.2-14.5)	0.416
	White	47.7 (39.4-56.2)		67.4 (57.2-76.2)		25.4 (19.7-32.0)		6.7 (3.3-13.4)		13.8 (6.8-26.2)	
	Coloured	44.0 (30.1-58.9)		59.3 (46.9-70.6)		11.2 (4.4-25.8)		5.1 (2.6-9.5)		25.6 (11.4-48.0)	
	Indian/Asian	32.0 (21.9-44.2)		26.1 (19.1-34.6)		6.5 (3.9-10.6)		0		13.4 (5.6-28.7)	
**Education**	None	40.8 (21.9-62.8)	0.152	55.9 (34.5-75.3)	0.574	21.6 (5.6-56.4)	0.020	0	0.094	11.6 (4.0-29.4)	0.482
	Grade 1-7	35.8 (28.3-44.2)		37.7 (29.7-46.5)		11.9 (7.0-19.4)		2.0 (0.6-6.8)		19.7 (12.3-29.9)	
	Grade 8-11	26.9 (23.2-31.0)		38.2 (33.8-42.8)		10.4 (7.9-13.6)		3.5 (1.9-6.5)		18.8 (14.7-23.6)	
	Matric	25.1 (20.7-30.0)		37.9 (33.3-41.8)		7.6 (5.3-10.7)		1.1 (0.5-2.7)		21.3 (16.5-27.1)	
	Matric +	29.5 (23.0-37.1)		40.3 (33.1-48.1)		16.8 (12.4-22.5)		4.3 (2.4-7.8)		24.3 (18.2-31.6)	
**Marital status**	Not married	25.9 (23.0-29.1)	0.073	38.8 (34.5-42.8)	0.684	10.0 (8.0-12.5)	0.289	2.7 (1.7-4.2)	0.978	19.8 (16.6-23.4)	0.241
	Married	31.9 (26.6-37.7)		37.3 (31.4-43.5)		12.8 (8.8-18.2)		2.6 (1.3-5.4)		23.1 (17.7-29.6)	
**Employment**	Employed	40.9 (33.8-48.4)	<0.001	56.1 (49.6-62.4)	<0.001	16.9 (12.5-22.4)	0.003	4.1 (2.4-7.1)	0.120	25.9 (19.3-33.9)	0.061
	Unemployed	23.9 (21.2-26.8)		33.8 (30.3-37.5)		9.1 (7.1-11.5)		2.3 (1.4-3.8)		19.2 (16.1-22.8)	
**Income** c	Zero	23.2 (19.3-32.1)	0.912	39.9 (31.9-48.5)	0.715	12.3 (7.7-19.2)	0.167	3.7 (1.2-11.2)	0.401	19.4 (13.5-27.2)	0.404
	Low	26.6 (31.7-32.1)		36.1 (31.7-40.6)		8.4 (6.0-11.7)		3.3 (1.7-6.6)		21.6 (17.6-26.3)	
	Low-average	28.5 (23.2-34.6)		42.5 (35.6-49.6)		7.3 (4.5-11.7)		0.6 (0.1-4.4)		15.8 (11.1-22.1)	
	High-average	28.7 (23.2-34.9)		37.6 (31.1-44.6)		12.4 (8.7-17.4)		3.5 (1.6-7.8)		20.5 (15.4-26.8)	
	High	28.2 (22.4-34.8)		38.3 (31.4-45.8)		13.0 (9.3-17.9)		1.8 (0.7-4.4)		23.1 (17.7-29.6)	
**Area of residence**	Rural	22.3 (17.9-27.4)	0.006	31.8 (26.5-37.7)	0.003	13.6 (11.0-16.8)	<0.001	1.7 (0.8-3.6)	0.133	15.8 (12.3-20.1)	0.010
	Urban	30.8 (27.9-33.8)		42.7 (38.9-46.6)		6.2 (4.7-8.2)		3.3 (2.2-5.2)		23.8 (19.5-28.7)	

a Cocaine, heroin, opium, glue, LSD, peyote

b Non-medical use of sedatives, tranquilisers, stimulants, analgesics

c Personal earnings from employment in the past 12 months, before taxes, excluding pensions and investments

**Table 2:** Prevalence of anxiety and mood disorders by substance use

		TOTAL	Tobacco Use		Alcohol Use		Cannabis		Other substances^a^		Non-medical use of prescription drugs b	
		Prevalence (95% CI)	100+ cigarettes% (CI)	p-value	Ever% (CI)	p-value	Ever% (CI)	p-value	Ever% (CI)	p-value	Ever% (CI)	p-value
Lifetime DSM-IV Disorders												
Anxiety Disorders	Panic Disorder	0.7 (0.3-1.3)	1.1 (0.4-2.8)	0.211	1.0 (0.4-2.3)	0.287	0.8 (0.1-5.8)	0.815	1.8 (0.2-12.1)	0.321	2.2 (1.0-5.1)	<0.001
	GAD with hierarchy	1.1 (0.7-1.7)	1.7 (0.9-3.5)	0.102	1.3 (0.6-2.5)	0.485	2.4 (0.9-6.4)	0.080	1.9 (0.3-13.3)	0.536	1.7 (0.7-4.0)	0.189
	Social Phobia	2.8 (2.0-4.1)	5.1 (2.7-9.6)	0.011	4.4 (2.4-8.1)	0.044	5.9 (2.7-12.5)	0.021	5.8 (0.8-33.4)	0.438	4.1 (2.0-8.3)	0.120
	Agoraphobia	11.1 (9.2-13.4)	10.0 (6.8-14.5)	0.498	12.2 (9.1-16.1)	0.387	8.8 (5.5-13.7)	0.276	4.0 (0.9-16.5)	0.142	10.8 (8.1-14.3)	0.816
	PTSD	4.5 (1.0-2.2)	2.1 (1.0-4.4)	0.230	2.6 (1.5-4.5)	0.004	5.1 (2.0-12.2)	0.003	10.6 (3.9-26.1)	<0.001	1.3 (0.4-3.6)	0.758
	Any anxiety disorder	14.7 (12.5-17.2)	16.4 (11.8-22.2)	0.388	17.7 (13.5-22.9)	0.063	17.7 (10.9-27.4)	0.405	24.1 (9.9-48.1)	0.219	16.4 (12.7-20.9)	0.312
Mood Disorders	Major Depression	8.6 (7.1-10.3)	12.1 (8.4-17.1)	0.027	11.8 (9.0-15.5)	0.001	15.4 (10.1-22.7)	0.003	27.2 (10.8-53.6)	0.009	12.8 (9.1-17.7)	0.010
Any anxiety or mood disorder		19.7 (17.3-22.3)	23.3 (18.0-29.8)	0.092	24.1 (19.7-19.1)	0.007	26.8 (19.4-35.8)	0.054	42.5 (21.0-67.3)	0.021	24.4 (19.8-19.6)	0.027
12-month DSM-IV Disorders												
Anxiety Disorders	Panic Disorder	0.5 (0.2-1.1)	1.1 (0.4-2.8)	0.068	0.7 (0.3-1.9)	0.415	0.8 (0.1-5.8)	0.594	1.8 (0.2-12.1)	0.199	2.2 (1.0-5.1)	<0.001
	GAD with hierarchy	0.7 (0.4-1.3)	1.6 (0.8-3.4)	0.005	1.2 (0.6-2.5)	0.062	2.4 (0.9-6.4)	0.007	1.9 (0.3-13.3)	0.295	0.9 (0.3-2.8)	0.691
	Social Phobia	1.9 (1.3-2.8)	2.9 (1.2-6.5)	0.192	2.7 (1.3-5.3)	0.189	4.3 (1.6-10.5)	0.042	4.8 (0.5-35.8)	0.361	3.3 (1.5-7.1)	0.067
	Agoraphobia	5.1 (3.9-6.6)	4.9 (2.9-8.2)	0.872	4.9 (3.1-7.9)	0.898	5.0 (2.5-9.8)	0.958	0	-	4.1 (2.8-6.0)	0.259
	PTSD	0.3 (0.2-0.7)	5.4 (0.2-1.9)	0.296	0.6 (0.2-1.6)	0.030	1.4 (0.4-4.9)	0.006	1.9 (0.3-12.8)	0.055	0	-
	Any anxiety disorder	7.1 (5.5-9.0)	8.8 (5.8-13.2)	0.102	8.1 (5.3-12.3)	0.340	10.1 (5.5-17.9)	0.181	10.4 (2.5-34.3)	0.532	8.1 (5.3-12.1)	0.384
Mood Disorders	Major Depression	4.3 (3.3-5.6)	7.0 (4.4-10.9)	0.037	6.1 (4.3-8.7)	0.012	11.0 (6.9-17.2)	<0.001	21.0 (7.2-47.5)	0.002	6.7 (4.2-40.7)	0.059
Any anxiety or mood disorder		10.3 (8.5-12.5)	14.7 (10.7-19.8)	0.004	12.9 (9.9-16.8)	0.019	19.6 (13.3-27.7)	0.001	26.6 (10.5-52.6)	0.025	12.9 (9.6-17.2)	0.086

a Cocaine, heroin, opium, glue, LSD, peyote

b Non-medical use of sedatives, tranquilisers, stimulants, analgesics

Note: GAD= generalised anxiety disorder; PTSD= post-traumatic stress disorder

**Table 3:** Crude associations between anxiety and mood disorders, and substance use, using regression analyses

		Tobacco Use		Alcohol Use		Cannabis		Other substances^a^		Non-medical use of prescription drugs b	
		OR (95% CI)	p-value	OR (95% CI)	p-value	OR (95% CI)	p-value	OR (95% CI)	p-value	OR (95% CI)	p-value
**Lifetime DSM-IV Disorders**											
Anxiety Disorders	Panic Disorder	2.5 (0.6-8.4)	0.223	2.1 (0.5-8.3)	0.298	1.3 (0.2-11.0)	0.815	2.8 (0.3-25.1)	0.342	9.3 (2.4-35.5)	0.001
	GAD with hierarchy	2.2 (0.8-5.8)	0.110	1.4 (0.6-3.4)	0.487	2.7 (0.8-8.9)	0.093	1.9 (0.2-15.6)	0.543	1.9 (0.7-5.2)	0.196
	Social Phobia	2.7 (1.2-5.8)	0.014	2.5 (1.0-6.0)	0.050	2.5 (1.1-5.4)	0.025	2.2 (0.3-16.5)	0.449	1.7 (0.9-3.2)	0.124
	Agoraphobia	0.9 (0.5-1.4)	0.499	1.2 (0.8-1.8)	0.388	0.7 (0.4-1.3)	0.278	0.3 (0.1-1.6)	0.162	1.0 (0.7-1.4)	0.816
	PTSD	1.8 (0.7-4.6)	0.236	3.8 (1.5-9.7)	0.007	5.2 (1.6-16.8)	0.006	9.8 (2.9-32.5)	<0.001	0.8 (0.2-2.8)	0.758
	Any anxiety disorder	1.2 (0.8-1.8)	0.388	1.5 (1.0-2.2)	0.064	1.3 (0.7-2.3)	0.406	1.9 (0.7-5.3)	0.226	1.2 (0.9-1.6)	0.312
Mood Disorders	Major Depression	1.8 (1.1-2.9)	0.029	1.9 (1.3-2.8)	0.001	2.2 (1.3-3.6)	0.003	4.3 (1.3-13.7)	0.016	1.8 (1.2-2.9)	0.011
Any anxiety or mood disorder		1.4 (0.9-1.9)	0.092	1.6 (1.1-2.1)	0.008	1.6 (1.0-2.5)	0.056	3.2 (1.1-8.7)	0.028	1.4 (1.0-1.9)	0.027
**12-month DSM-IV Disorders**											
Anxiety Disorders	Panic Disorder	4.1 (0.8-20.8)	0.091	1.9 (0.4-9.2)	0.423	1.8 (0.2-16.1)	0.599	3.8 (0.4-35.7)	0.232	49.5 (9.4-259.4)	<0.001
	GAD with hierarchy	4.5 (1.4-13.8)	0.010	2.9 (0.9-9.0)	0.074	4.9 (1.4-17.3)	0.015	2.9 (0.3-24.8)	0.318	1.3 (0.3-5.2)	0.691
	Social Phobia	1.9 (0.7-5.0)	0.199	1.9 (0.7-5.0)	0.196	2.7 (1.0-7.4)	0.050	2.7 (0.3-26.6)	0.381	2.2 (0.9-5.0)	0.073
	Agoraphobia	1.0 (0.6-1.6)	0.872	1.0 (0.5-1.8)	0.898	1.0 (0.6-2.1)	0.958	-	-	0.8 (0.5-1.2)	0.260
	PTSD	2.3 (0.5-11.3)	0.309	4.6 (1.0-20.7)	0.047	7.2 (1.4-36.3)	0.017	6.8 (0.7-64.5)	0.096	-	-
	Any anxiety disorder	1.4 (0.9-2.2)	0.104	1.3 (0.7-2.3)	0.341	1.6 (0.8-3.1)	0.184	1.6 (0.4-6.4)	0.535	1.2 (0.8-1.9)	0.385
Mood Disorders	Major Depression	2.2 (1.1-4.6)	0.034	2.0 (1.2-3.5)	0.013	3.4 (1.9-6.4)	<0.001	6.7 (1.7-25.6)	0.007	1.9 (1.0-3.8)	0.063
Any anxiety or mood disorder		1.8 (1.2-2.7)	0.004	1.6 (1.1-2.3)	0.019	2.4 (1.4-4.0)	0.001	3.3 (1.1-9.8)	0.033	1.9 (1.0-2.0)	0.087

a Cocaine, heroin, opium, glue, LSD, peyote

b Non-medical use of sedatives, tranquilisers, stimulants, analgesics

Note: GAD= generalised anxiety disorder; PTSD= post-traumatic stress disorder

**Table 4:** Adjusted associations between anxiety and mood disorders and substance use using regression analyses

		Tobacco Use		Alcohol Use		Cannabis		Other substances^a^		Non-medical use of prescription drugs b	
		OR (95% CI)	p-value	OR (95% CI)	p-value	OR (95% CI)	p-value	OR (95% CI)	p-value	OR (95% CI)	p-value
**Lifetime DSM-IV Disorders**											
Anxiety Disorders	Panic Disorder	3.1 (0.7-13.7)	0.129	2.5 (0.8-7.8)	0.0124	1.4 (0.2-11.3)	0.731	3.1 (0.3-33.5)	0.349	9.5 (2.5-36.5)	0.001
	GAD with hierarchy	3.0 (1.0-9.4)	0.061	1.5 (0.6-3.8)	0.373	3.2 (0.9-12.2)	0.082	2.0 (0.2-17.6)	0.516	1.87 (0.7-5.1)	0.214
	Social Phobia	3.9 (1.5-10.1)	0.006	3.0 (1.1-7.9)	0.030	2.9 (1.1-7.5)	0.025	2.3 (0.3-21.6)	0.460	1.7 (0.9-3.4)	0.113
	Agoraphobia	1.2 (0.8-2.1)	0.390	1.6 (1.0-2.5)	0.044	1.0 (0.6-1.8)	0.997	0.4 (0.1-2.0)	0.266	1.0 (0.7-1.4)	0.966
	PTSD	2.7 (0.8-9.1)	0.119	5.2 (2.0-13.4)	0.001	8.1 (2.3-29.2)	0.002	12.5 (3.9-40.6)	<0.001	0.8 (0.2-2.8)	0.712
	Any anxiety disorder	1.8 (1.1-2.9)	0.011	2.0 (1.3-3.2)	0.004	1.7 (0.9-3.3)	0.087	2.4 (0.8-6.8)	0.108	1.2 (0.9-1.7)	0.247
Mood Disorders	Major Depression	3.0 (1.7-5.4)	<0.001	2.7 (1.8-3.9)	<0.001	3.1 (1.8-5.5)	<0.001	5.6 (17-18.1)	0.005	1.8 (1.2-2.0)	0.011
Any anxiety or mood disorder		2.2 (1.4-3.2)	<0.001	2.1 (1.5-3.1)	<0.001	2.2 (1.3-3.6)	0.003	4.0 (1.5-11.1)	0.008	1.5 (1.1-2.0)	0.020
**12-month DSM-IV Disorders**											
Anxiety Disorders	Panic Disorder	6.9 (1.5-31.7)	0.014	2.3 (0.6-8.5)	0.215	2.1 (0.3-16.3)	0.471	4.3 (0.4-52.8)	0.247	50.5 (9.5-268.4)	<0.001
	GAD with hierarchy	6.2 (1.5-25.7)	0.012	3.1 (0.9-10.3)	0.065	5.5 (1.2-25.4)	0.028	2.9 (0.3-27.6)	0.356	1.3 (0.3-5.1)	0.723
	Social Phobia	3.5 (1.0-12.9)	0.059	2.7 (0.9-8.0)	0.066	4.4 (1.5-13.0)	0.009	3.7 (0.3-42.6)	0.296	2.2 (1.0-5.1)	0.063
	Agoraphobia	1.9 (1.1-3.1)	0.016	1.5 (0.7-3.0)	0.289	1.7 (0.7-3.8)	0.216	-	-	0.8 (0.5-1.3)	0.356
	PTSD	3.1 (0.4-24.2)	0.276	5.9 (1.3-27.6)	0.025	10.4 (1.5-70.1)	0.017	7.5 (0.7-75.2)	0.086	-	-
	Any anxiety disorder	2.9 (1.8-4.6)	<0.001	2.0 (1.0-3.8)	0.043	2.6 (1.2-5.6)	0.012	2.2 (0.5-9.9)	0.284	1.3 (0.8-2.0)	0.302
Mood Disorders	Major Depression	4.7 (2.2-10.0)	<0.001	3.0 (1.7-5.4)	<0.001	6.3 (3.0-13.2)	<0.001	10.2 (2.8-37.0)	0.001	1.9 (1.0-3.8)	0.060
Any anxiety or mood disorder		3.8 (2.5-5.8)	<0.001	2.4 (1.5-3.7)	<0.001	4.1 (2.3-7.4)	<0.001	4.7 (1.6-14.4)	0.006	1.4 (1.0-2.1)	0.058

^†^ Adjusted for age and gender

a Cocaine, heroin, opium, glue, LSD, peyote

b Non-medical use of sedatives, tranquilisers, stimulants, analgesics

Note: GAD= generalised anxiety disorder; PTSD= post-traumatic stress disorder

Basic sociodemographic information was elicited, including both time-fixed (age, gender and RCSG) and time-varying factors (highest educational level, marital status, employment, income and area of residence). Income was defined as the participant's personal earnings from employment in the past 12 months, before taxes, and excluded income from investments or pensions. Participants also answered questions regarding their own substance use, including use of tobacco; alcohol; cannabis; cocaine; LSD; heroin; opium; glue; and non-medical use of sedatives, tranquilisers, stimulants, analgesics, or any other psychoactive over-the-counter compounds. Tobacco smokers were defined as those who reported having smoked more than 100 cigarettes in their lifetime. Alcohol use was defined as ever having had a drink of alcohol. Use of other drugs was defined as ever having used the specific drug.

### Data analyses

Data were analysed using Stata Version 11.0 [34], and considered the survey design based on person-level weights, and incorporated sample selection, non-response and post-stratification factors. All statistical tests were two-sided at a = 0.05.

Descriptive statistics were generated, providing mean age, and proportions for categorical data. The prevalence of the demographic and socioeconomic variables was examined and stratified, and presented by substance use categories (tobacco, alcohol, cannabis, other drugs, extra-medical drugs). Differences between the proportions were tested with the chi-squared test, and p-values reported to compare demographic and socioeconomic characteristics by substance use. The prevalence and 95% confidence intervals of lifetime and 12-month DSM-IV disorders were stratified by substance use and p-values from chi-squared tests for proportions were reported, with prevalence of comorbidity reported for specific forms of psychopathology in relation to specific substances of use. To examine associations between DSM-IV disorders (both lifetime and 12-month) and substance use, regression analyses were conducted, including adjustment for age (as a continuous variable) and gender.

## Results

The study sample consisted of 1766 males and females, comprising a subset of the SASH survey sample (n = 4351), and consisting of all the SASH survey participants aged 18 to 30 years (Mean age 23.6 years; 95% CI 23.4-23.8).

Table 1 summarises the prevalence of substance use for the selected demographic factors. This table indicates that 27.4% of the sample had smoked 100 or more cigarettes in their lifetime, 38.7% had ever used alcohol, 10.7% had ever used cannabis, 2.7% had used other substances such as cocaine, heroin, opium, glue, LSD or peyote, and 20.6% had used sedatives, tranquilisers, stimulants or analgesics for non-medical use. Significantly more males than females smoked cigarettes (p < 0.001) and significantly fewer Blacks smoked cigarettes compared with other RCSGs (p < 0.001). Smokers were also more likely to be employed (p < 0.001) and to live in urban areas (p = 0.006).

A larger proportion of males used alcohol compared with females (p < 0.001). A significantly smaller proportion of Indians/Asians used alcohol compared with the other RCSGs (p < 0.001). Significantly more alcohol users were employed (p < 0.001), and lived in urban areas (p = 0.003). The proportion of cannabis users differed significantly by gender (p < 0.001), RCSG (p < 0.001), educational level (p = 0.020), employment status (p = 0.003) and residential area (p < 0.001). Significantly more males than females used cannabis (p < 0.001). A larger proportion of Whites had ever used cannabis compared with the other RCSGs. The highest proportions of cannabis users were reported for those who had no formal education (21.6%) and those who had tertiary education (16.8%), suggesting a bimodal distribution. A significantly larger proportion of cannabis users were unemployed and lived in rural areas.

A larger proportion of males had ever used other drugs (p < 0.001) while the proportion of Blacks who used these substances was significantly smaller than the proportions of other RCSGs who used these substances (p = 0.004). A significantly larger proportion of individuals who reported non-medical use of prescription drugs lived in urban areas (p = 0.010) compared with the proportion who lived in rural areas.

Overall comorbidity rates in the SASH sample were as follows (percentages adjusted for survey weighting with 95% CIs): For any lifetime substance use and any DSM IV disorder 21.3% (CI 18.8-23.9); for any lifetime substance use disorder and any anxiety or depression disorder 4.0% (CI 2.6-6.1); for any 12-month substance use and any DSM IV disorder 11.5% (CI 9.2-14.2); for any 12-month substance use disorder and any anxiety or depression disorder 1.6% (CI 1.0-2.7).

Table 2 lists the odds ratios for mental disorders by substance of use compared with absence of the particular substance of use. In the total sample, the odds of any lifetime anxiety disorder were 14.7, and 19.7 for any lifetime anxiety or mood disorder. The odds of any 12-month anxiety or mood disorder were 10.3 while for the total sample, the odds of having any 12-month anxiety disorder was 7.1. The odds ratios of lifetime anxiety disorders was lowest for panic disorder (0.7) and highest for agoraphobia(11.1), while the odds ratios of 12-month anxiety disorders was lowest for PTSD (0.3) and highest for agoraphobia (5.1). The odds ratios were 8.6 for lifetime major depression, and 4.3 for 12-month major depression.

Compared with non-tobacco users, tobacco users had a significantly higher likelihood of lifetime social phobia (OR = 5.1; p = 0.011), and major depression (OR = 12.1; p = 0.027), and had significantly higher odds of 12-month generalised anxiety disorder (OR = 1.6; p = 0.005), major depression (OR = 7.0; p = 0.037) and any anxiety or mood disorder (OR = 14.7; p = 0.004). Alcohol users differed significantly from non-alcohol users with respect to the prevalence of lifetime social phobia, PTSD, major depression and any anxiety or mood disorder. Alcohol users also differed significantly from non-alcohol users with respect to the prevalence of 12-month PTSD, major depression and any anxiety or mood disorder. Those who had ever used cannabis differed significantly from those who had never used cannabis with respect to the prevalence of lifetime social phobia, PTSD, major depression, and 12-month generalised anxiety disorder, PTSD, major depression and any anxiety or mood disorder. Users of other drugs differed significantly from non-users with respect to the prevalence of lifetime PTSD, major depression and any anxiety or mood disorder, and to 12-month major depression and any anxiety or mood disorder. The proportion of individuals who reported non-medical use of prescription drugs differed significantly from non-users with respect to the prevalence of lifetime panic disorder, major depression and any anxiety or mood disorder, and 12-month panic disorder.

Table 3 presents associations between any anxiety or mood disorders and substance, while Table 4 presents associations adjusted for age and gender. It is evident from Table 3 and Table 4 that the odds of comorbid psychopathology are increased in the presence of substance use compared with the odds of psychopathology in the absence of substance use. For example, the odds of having any lifetime anxiety or mood disorder was 1.4 with use of tobacco and non-medical use of prescription drugs, 1.6 with use of alcohol or cannabis, and 3.2 with use of other substances (Table 3). The equivalent odds on adjustment for age and gender were 2.2 and 1.5 for tobacco use and non-medical use of prescription drugs respectively, 2.1 and 2.2 for alcohol and cannabis use, and 4.0 for other substance use (Table 4). Similarly, the odds of a 12-month mood or anxiety disorder, on adjustment for age and gender, were 1.4 for non-medical use of prescription drugs, 2.4 for alcohol use, 3.8 for tobacco use, and 4.1 and 4.7 for cannabis and other substance use respectively (Table 4).

On adjustment for age and gender, there was approximately a twofold increased likelihood of lifetime psychopathology, and a threefold increased likelihood of 12-month psychopathology in tobacco users versus non-tobacco users. Tobacco users had a six- sevenfold increased likelihood of 12-month panic or generalised anxiety disorder over non-tobacco users, and a 5-fold increased likelihood of 12-month major depression. The increased likelihood of lifetime and 12-month PTSD in alcohol, cannabis and other drug users, the increased likelihood of 12-month major depression in cannabis and other drug users, and the increased likelihood of lifetime and 12-month panic disorder in individuals who reported non-medical use of prescription drugs, were significant.

On adjustment for age and gender, lifetime generalised anxiety disorder (GAD) was not significantly associated with any of the substances of use examined, while lifetime agoraphobia, panic disorder, PTSD and social phobia were associated with only some of the selected substances of use. Similarly, with regards to 12-month disorders, the specific disorders were significantly associated with certain, and not all, the selected substances of use. Both lifetime and 12-month major depression were significantly associated with all (barring one) the selected substances of use.

## Discussion

The main findings of the study were that, in young adults 1) substance users were more likely than non-substance users to be male, White, employed and living in urban environments, 2) lifetime and 12-month anxiety or mood disorder was more likely to occur in substance users than in non-users of substances, and 3) strengths of association between psychopathology and substances of use were higher in relation to specific psychopathology (for example PTSD) and specific substances of use (for example, cannabis and alcohol).

Overall, the results obtained concur with previous findings that males were more likely than females to smoke [19], and to have been regular users of alcohol [35] and other drugs. These results possibly reflect the stereotypical norms of male socialisation with regards to cigarette smoking and alcohol use. However, recent evidence suggests that, as traditional gender roles start to equalise, prevalence of female substance use might approach that of males, with potentially similar consequences regarding substance-related morbidity [36].

Employed individuals were more likely to use substances, possibly reflecting greater access to disposable income, compared with unemployed individuals. However, substance users did not differ significantly from non-substance users with respect to income, thus suggesting that factors other than economic resources and linked to employment status, such as access to particular social networks, may also be operative.

Blacks were the least likely of the RCSGs to smoke cigarettes, potentially suggesting a protective factor(s) against cigarette smoking among the Blacks in this sample, or an increased rate of cigarette smoking in the non-Black population, or a link between being Black and being unemployed. Similarly, individuals of Indian or Asian origin were the least likely to use alcohol, cannabis or any substances other than cigarettes. These findings concur with that of studies reported by Rodriguez et al. [37] who found associations between unemployment and substance abuse, but also demonstrated differences between population groups in terms of the impact that factors such as gender, marital status, employment status, job satisfaction and educational level had on health and wellbeing. Other reports from the SASH study showed that there were marked racial differences on all indicators of economic status including education, income, employment, and ownership of material resources [38]. The latter authors suggested that it might be important to distinguish between minority status and racial-ethnic groups when assessing the impact of sociodemographic factors on the mental health of individuals in South Africa.

Substance users in the SASH sample were more likely than non-substance users to have had lifetime or 12-month anxiety disorders or major depression, irrespective of the choice of substance use. In addition, the results in the current study indicated statistically significant associations between psychopathology and substance use, irrespective of the substances of use. These findings echo those of several earlier studies reviewed by Saban and Flisher [11] that indicated increased risk of psychiatric disorder with substance use [11]. The trends in this younger adult sample are also similar to those in the National Comorbidity Survey- Replication (NCS-R), with the prevalence of the anxiety disorders exceeding that of the mood disorders [39]. Further, these results similarly concur with the findings of the National Epidemiologic Survey on Alcohol and Related Conditions (NESARC) [40].

The results also indicate that illicit substances such as cannabis have a manifold increased risk of psychopathology with adjustment for age and gender. The Australian National Survey of Mental Health and Well-being (NSMHWB) reported similar findings in relation to adult anxiety disorders [28]. It is thus important that the risks of psychopathology with cannabis use be emphasised, particularly in communities where exposure to cannabis use is high. It is also important that the factors that place individuals at risk of cannabis use are identified and addressed, both to protect against cannabis use, and to decrease the risk of associated psychopathology.

The results indicated relatively high increased odds of lifetime and 12-month PTSD, particularly when PTSD was comorbid with alcohol or cannabis. Earlier studies with substance-using inpatients and war veterans also provided evidence for a strong association between PTSD and substance use and related disorders [41, 42]. However, these studies have cautioned that the associations be considered with particular reference to the role of trauma in more general family or social dysfunction. Thus, it might be necessary to identify the factors that predispose individuals with PTSD diagnoses to substance use, and particularly alcohol and cannabis use, and to identify the factors that increase the risk of PTSD in substance users [43].

Several limitations of the SASH study should be recognised when interpreting these results. Firstly, the SASH sample excluded individuals who were in prison, hospital or mental institutions, or who lived on military bases. Thus psychiatric disorders or substance use pertinent to these categories of individuals would have been underestimated or excluded from the sample. These include, for example, antisocial personality disorders and available substances of use in the prison population, psychiatric or substance use diagnoses that might have precipitated admission in hospital or mental institution patients, and PTSD that might have been more prevalent in soldiers than in the non-military community. Secondly, the SASH survey assumed equal chance of representation of mentally ill and healthy subjects in the sample, even though individuals with psychopathology are known to be less likely than those without psychopathology to be willing participants in surveys, particularly when those surveys relate to mental illness [44] (citing Kessler, Wittchen and Abelson, 1998). These are factors that might have skewed the prevalence of psychopathology in favour of mentally-healthy individuals, and provided an under-estimate of substance use. Thirdly, the SASH data excluded psychiatric diagnoses such as bipolar disorder, oppositional defiant disorder, conduct disorder, attention deficit hyperactivity disorder, obsessive compulsive disorder, specific phobia and separation anxiety disorder, thus limiting the associations between psychopathology and substance use that could be examined. Fourthly, the cross-sectional nature of the sample did not permit identification of causative factors in the associations, or the temporal order of co-occurring psychopathology and substance use. Fifthly, the SASH data relied on self-reports. The reliability of the information obtained might thus have been compromised if, for example, use of substances was under- or over-reported, particularly since this information was not verified with biomarkers. Lastly, the statistical analyses for this study involved multiple comparisons between variables, suggesting that the statistically significant results might have been chance findings and, thus, subject to error. This error could have benefitted from adjustment using the Dunn-Bonferroni correction. However, this correction was not applied here since an inherent limitation of the method is to increase the probability of false negative results, thus reducing statistical power in the study.

## Conclusion

The results obtained highlight the prevalence of substance use in young adults, with particular reference to sociodemographic factors and the most commonly-occurring psychiatric diagnoses, and have provided compelling evidence for an association between psychopathology and substance use.
